# The UK Pharmacy Care Plan service: Description, recruitment and initial views on a new community pharmacy intervention

**DOI:** 10.1371/journal.pone.0174500

**Published:** 2017-04-03

**Authors:** Michael J. Twigg, David Wright, Charlotte L. Kirkdale, James A. Desborough, Tracey Thornley

**Affiliations:** 1 School of Pharmacy, University of East Anglia, Norwich Research Park, Norwich, United Kingdom; 2 Boots UK, Beeston, Nottingham, United Kingdom; 3 School of Pharmacy, University of Nottingham, Nottingham, United Kingdom; Brown University, UNITED STATES

## Abstract

**Introduction:**

The UK government advocates person-centred healthcare which is ideal for supporting patients to make appropriate lifestyle choices and to address non-adherence. The Community Pharmacy Future group, a collaboration between community pharmacy companies and independents in the UK, introduced a person-centred service for patients with multiple long-term conditions in 50 pharmacies in Northern England.

**Objective:**

Describe the initial findings from the set up and delivery of a novel community pharmacy-based person-centred service.

**Method:**

Patients over fifty years of age prescribed more than one medicine including at least one for cardiovascular disease or diabetes were enrolled. Medication review and person-centred consultation resulted in agreed health goals and steps towards achieving them. Data were collated and analysed to determine appropriateness of patient recruitment process and quality of outcome data collection. A focus group of seven pharmacists was used to ascertain initial views on the service.

**Results:**

Within 3 months of service initiation, 683 patients had baseline clinical data recorded, of which 86.9% were overweight or obese, 53.7% had hypertension and 80.8% had high cardiovascular risk. 544 (77.2%) patients set at least one goal during the first consultation with 120 (22.1%) setting multiple goals. A majority of patients identified their goals as improvement in condition, activity or quality of life. Pharmacists could see the potential patient benefit and the extended role opportunities the service provided. Allowing patients to set their own goals occasionally identified gaps to be addressed in pharmacist knowledge.

**Conclusion:**

Pharmacists successfully recruited a large number of patients who were appropriate for such a service. Patients were willing to identify goals with the pharmacist, the majority of which, if met, may result in improvements in quality of life. While challenges in delivery were acknowledged, allowing patients to identify their own personalised goals was seen as a positive approach to providing patient services.

## Introduction

Whilst the concept of person-centred care, where patients set their own health goals is well accepted [1–4], there has been a recent increase in focus on the topic within the UK National Health Service (NHS) [5, 6]. The United Kingdom (UK) Government has stated that patients should be actively involved in their care with the concept of shared decision making at the heart of NHS services and putting patients’ needs at the centre of any intervention they are undergoing [7]. Key aspects involved in a person-centred approach include listening to the individual to understand their perspective, providing information in a manner which enables the person to make informed decisions and supporting them to develop goals relating to their lifestyle, health and medicines [5–7].

In England, coronary heart disease (CHD) results in 100,000 deaths per annum with 1.5 million suffering from angina and 275,000 suffering heart attacks [8]. However, up to 50% of cardiovascular deaths may be prevented through interventions which target modifiable risk factors such as hypertension, cholesterol, smoking, obesity and physical exercise [8]. People displaying some of these risk factors are also more likely to experience other co-morbidities e.g. type 2 diabetes, asthma and chronic back pain [9].

NHS statistics suggest that many patients do not achieve health related targets for CHD [10, 11] and this is due to sub-optimal prescribing, non-adherence to treatment and inappropriate lifestyle choices not being addressed with support from healthcare professionals. A review relating to cardiovascular patients demonstrated that only 63% of patients continued with their medication after one year [12]. Evidence also demonstrates that only 16% of patients prescribed a new medicine take it as prescribed, experience no problems and receive as much information as they need, whilst ten days after starting a medicine, almost one-third of patients are non-adherent [13].

With a need to prescribe according to evidence based guidelines [14–16], to increase the likelihood of patients taking their medicines as agreed with the prescriber [17] and provide support to address inappropriate lifestyle choices, the community pharmacist, who will regularly supply medicines to such patients, is ideally located to provide such a service. The specialist knowledge held by community pharmacists can be used to improve pharmaceutical care whilst a person-centred approach is ideal for addressing non-adherence and supporting patients to make appropriate lifestyle choices.

Person-centred care can be provided by community pharmacists when delivering interventions to improve public health such as smoking cessation clinics, weight management clinics and sexual health services. It can also be used to structure adherence focussed services relating to the initiation of new medicines and medicines use reviews. The appearance of consultation rooms in over 95% of community pharmacies in the UK [18] means that extended consultations can now be undertaken in a private environment which allows the pharmacist to focus purely on the patient in front of them.

The application of person-centred approaches is however currently untested in community pharmacy and potential enablers, barriers and outcomes are unknown. This is also the first UK community pharmacy service to provide such comprehensive care over a long period. Therefore, there is a need to learn from the implementation of a new person-centred community pharmacy service and to ascertain its feasibility.

This paper will focus on describing a new person-centred community pharmacy service, reporting initial recruitment and consultation data and pharmacist opinions regarding implementation and delivery of such an approach.

## Method

Approval for this service evaluation was provided by the Faculty of Medicines and Health Sciences Ethics Committee at the University of East Anglia (UEA) before commencement. Patients were asked to provide written consent to participate. This information was retained in the pharmacy.

### Setting

The Community Pharmacy Future (CPF) team is a partnership of four multiple pharmacy companies (Boots UK, LloydsPharmacy, Rowlands Pharmacy and Well) that came together to develop and implement new services in community pharmacies at their own expense. Fifty-two community pharmacies located in Northern England were asked by the CPF team to provide the service, with ten pharmacies selected from each of the four companies plus twelve invited that were either independent pharmacies or supermarkets. This location was chosen due to the active pharmacy primary care research team in the area, good working relationships with medical practices, broad representation of all CPF partners and is broadly representative of the UK.

Participating community pharmacists were encouraged to contact the medical practices most closely associated (in terms of prescription flow) with their pharmacy to highlight the service to them and engage with them at an early stage. A co-ordinated approach was used to engage medical practices associated with multiple participating pharmacies to avoid duplicating visits.

### Training

All community pharmacists completed an in-house CPF-developed training package which included a one day face-to-face training session for them and a member of their pharmacy support team on:

- Service specification
- Measurement of blood pressure and cholesterol
- Completion of the pharmacy care record and patient care plan
- Consultation skills

It was assumed that community pharmacists would be able to appropriately respond to any identified public health needs such as smoking cessation, weight loss and nutritional advice.

### Patient eligibility

Eligible patients were identified by the community pharmacy providing the service according to the criteria listed below:

#### Inclusion criteria

- Age > 50 years
- Patient prescribed more than one including one or more drugs from British National Formulary chapter 2 (cardiovascular) or 6.1 (diabetes)
- Consent to participate provided

#### Exclusion criteria

- Previously experienced a myocardial infarction, transient ischaemic attacks (TIA), angina or stroke. These patients were excluded as the measure of cardiovascular risk cannot be calculated in these patients.

**Fig 1:**
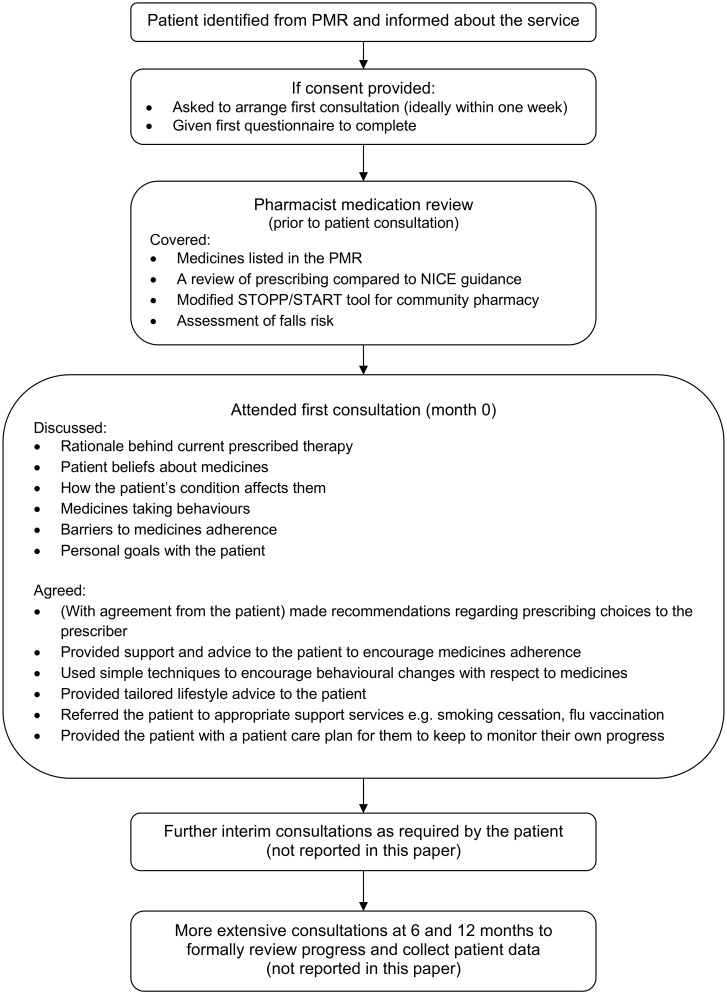
Patient flow through the PCP service.

The patient was identified from their pharmacy medication record (PMR) and when they presented to collect their dispensed prescription they were given information about the service. If they agreed to participate, they were asked to sign a consent form. An appointment was then arranged with the pharmacist, ideally within one week. Once consented, the patient was asked to complete the initial questionnaire which formed the basis of the first consultation. A diagrammatic representation of the service is shown in Fig 1. Clinical data (height, weight, blood pressure and cholesterol) for the service were collected by the healthcare assistant prior to meeting with the pharmacist.

### Patient questionnaire

The questionnaire used before the initial consultation, which was designed to be of a minimal length in order to maximise patient engagement in the service, contained the following measures:

- Quality of life measure (EuroQol EQ-5D-5L) [19]
- Patient reported medication adherence (Morisky MMAS-8) [20–22]
- Patient Activation Measure (PAM 10 item version), designed to measure patient knowledge, skills and confidence to manage their health [23]
- Four healthcare utilisation questions
- Two demographic questions regarding smoking and ethnicity

These measures will be further discussed in subsequent publications focussing on outcomes.

### Pharmacy Care Plan (PCP) service

The Pharmacy Care Plan (PCP) service was designed to have a holistic person centred focus and therefore not disease specific. The intervention which was focussed on patients prescribed multiple medications was designed to both enhance the effectiveness of the patient’s medicines and to improve lifestyle in order to improve their quality of life. The process involved an initial medication review followed by a consultation with the patient. Support staff in some pharmacies contributed to patient recruitment, organisation of appointments and data collection.

#### Medication review

Prior to meeting the patient, the pharmacist conducted an initial paper-based medication review from information held on the PMR. They reviewed prescribing using National Institute for Health and Care Excellence (NICE) guidance for the relevant conditions being treated [14–16]. For older patients (>65 years) the pharmacist used a modified STOPP/START tool [24] (appropriate for use in the community pharmacy setting and used in a previous service [25]) to enable them to structure their medication review. Additionally, they reviewed the risk/benefit of all medicines with respect to falls prevention.

#### Patient consultation

The medication review was followed by a face-to-face consultation with the patient and the joint production of a personalised care plan. At the first consultation the pharmacist or member of the pharmacy team obtained patient data to allow the calculation of the patient’s cardiovascular risk using the QRisk2 (2016) instrument [26]. The pharmacist then discussed and agreed the items listed in figure one to inform the development of a personalised patient care plan. The pharmacist supported patients to create their own personalised health goals and then agreed actions with the patient and plans for follow up and monitoring, including the use of validated tools to support the process [19, 20, 23]. The categorisation of these goals on the service database was open to interpretation by the pharmacist who could categorise any goal in a way that they felt was most appropriate. Any referrals to the patient’s GP were communicated using a standardised GP referral form which the pharmacist sent directly to the medical practice.

#### Further service provision

Post-initial consultation the pharmacist subsequently undertook brief consultations with the patient at regular intervals to discuss progress, provide further advice and conduct follow-up monitoring. This took place when the patient attended the pharmacy to obtain their dispensed repeat prescriptions or as necessary (nominally every two months). Two formal follow-up appointments were planned at six and twelve months after the initial meeting to monitor progress. The data from these interim, six and twelve month consultations will be analysed and reported in subsequent papers.

The initial consultation was predicted to last 40 minutes with subsequent consultations lasting from 15–25 minutes depending on whether these were interim or data collection consultations.

### Evaluation of pharmacists’ opinions

After the initial patient consultation a focus group was undertaken with pharmacists who were providing the PCP service to explore their thoughts on the intervention and how they had implemented it within their pharmacy.

Focus groups allow participants to share and clarify their own experiences by responding to others views leading to more detailed responses than survey methods [27]. This approach helped ensure a thorough exploration of pharmacist’s opinions within this study. The focus group was held at a CPF pharmacist feedback event in West Yorkshire in September 2015. A convenience sample of two pharmacists from each company were invited, as well as two independent pharmacists (due to pharmacist professional commitments). The focus group was led by a pharmacist qualitative researcher from UEA (MT), with experience in conducting interviews and focus groups, and was assisted by a non-pharmacist research manager from the CPF group (CK). Topics discussed were developed using the Theoretical Domains Framework (TDF) [28] and covered:

- Training provided for the service
- Recruitment and consent procedures
- Delivering the service in the context of their routine practice
- Staff involvement and interaction with other healthcare professionals
- Feedback for future service delivery

The focus group was audio recorded and transcribed verbatim. The transcription accuracy was checked by a second researcher.

### Data analysis

Data collected as part of the PCP service was entered by pharmacists on to a standard pharmacy service database used routinely in community pharmacy in the UK. Anonymised data were transferred to the UEA team from the service database for analysis.

#### Quantitative analysis

The data from the PCP service and completed questionnaires were collated and entered on separate SPSS databases (Statistical Software for Social Sciences version 22). Data were assessed for accuracy via visual, range and logic checks. Descriptive statistics were used to describe quantitative data: mean and standard deviation for interval data, median and interquartile ranges for ordinal or skewed interval data, and number and percent for nominal data.

#### Qualitative analysis

A framework analysis approach was used [29] which involved five key stages: familiarisation (listening to the interview, transcribing and reading transcripts), identifying a framework, indexing, charting, mapping, and interpretation. Analysis of the transcript involved indexing pharmacists’ speech according to one or more of the 14 TDF domains [28] and then grouping anonymised quotes from pharmacists into their relative TDF domains e.g. beliefs about consequences. This was first completed by three researchers and then discussed within the group to ensure interpretation was clear. Any discrepancies were highlighted and perceptions of the domains discussed to ensure the framework was being applied to the transcript consistently. The 14 TDF domains were used for further abstraction into overarching themes as the same passage of text was often included in multiple TDF domains.

## Results

### Patient data

Recruitment into the pharmacy care plan service (PCP) took place from the 2^nd^ February 2015 to 7^th^ June 2015. In total 866 patients expressed an interest in the service from 48 community pharmacies, resulting in a mean recruitment rate of 1.1 patients per pharmacy per week. Of these, 683 patients in 42 pharmacies had the first consultation with the pharmacist and completed the baseline questionnaire. The remaining 183 patients were assumed to have withdrawn from the service. Reasons for withdrawal were collected from 49/183 (26.8%) and included patients ‘not having time for the service’ (20/49), ‘not needing the service’ (10/49) and ‘not wanting to be bothered again’ (9/49). The mean (SD) age of patients at the first consultation was 67.7 (8.6) with 380 (55.6%) women participating in the service. The figures for the drop-outs were 67.0 (9.2) and 96 (52.5%) respectively. Of those participating in the first consultation, 665 (97.4%) were white, 427 (62.5) were non-smokers (never smoked) and 177 (25.9%) were ex-smokers.

Table 1 illustrates that the majority of patients recruited to the study were overweight or obese (86.9%), had hypertension, defined as blood pressure >140/90 mmHg, (53.7%) and a high (>20%) QRisk2 score (80.8%). In total, 683 (100%) patients completed the patient activation measure, MMAS-8 and the initial EQ-5D-5L assessment. Raw data is displayed in S1 Dataset.

**Table 1:** Clinical data for patients completing the initial stages of the PCP service.

Clinical measure (N = 683)		Measure	Result
**Weight (Kg)**		Mean (SD)	84.3 (18.6)
**BMI (Kg/m**^**2**^**)**	Underweight	N (%)	2 (0.3)
	Healthy	N (%)	87 (12.7)
	Overweight	N (%)	242 (35.4)
	Obese	N (%)	352 (51.5)
**Systolic BP (mmHg)**		Mean (SD)	140.5 (18.3)
**Diastolic BP (mmHg)**		Mean (SD)	78.8 (10.8)
**Hypertension (≥140/90mmHg)**		N (%)	367 (53.7)
**Total cholesterol (mmol/L)**		Mean (SD)	4.0 (1.2)
**HDL cholesterol (mmol/L)**		Mean (SD)	1.3 (0.6)
**QRisk2 score**		Mean (SD)	20.8 (13.1)
**High QRisk2 score**		N (%)	552 (80.8)
**Diagnosed conditions**	Diabetes*	N (%)	265 (38.8); 227 (33.2) type 2
	CKD	N (%)	10 (1.5)
	AF	N (%)	30 (4.4)
	RA	N (%)	72 (10.5)
	Angina in a 1^st^ degree relative <60	N (%)	104 (15.2)

BP: blood pressure; HDL: High density lipoprotein; CKD: chronic kidney disease; AF: atrial fibrillation; RA: rheumatoid arthritis;

*Both type 1 and type 2 diabetes.

In total, 544 (77.2%) patients set at least one goal during the first consultation with 120 (22.1%) setting multiple goals. The majority of the goals were categorised as wanting an improvement in activity or quality of life (35.5%), improvement in condition (27.9) and reduction in symptoms (9.6%). Table 2 provides greater detail on the type of goals agreed.

**Table 2:** Goals agreed as part of the service.

Goal category	N	% (of patients = 544)
**Improvement in activity or quality of life**	254	46.7
**Improvement in condition**	200	36.8
**Reduction in symptoms**	69	12.7
**Use of medication**	44	8.1
**Emotional improvement**	37	6.8
**Information seeking**	27	5.0
**Other:**	83	15.3
** Weight**	41	7.5
** Diet**	14	2.6
** None**	8	1.5
** Exercise**	7	1.3
** Smoking**	6	1.1
** Maintain**	6	1.1
** Other**	1	0.2
**Total**	**714**	

### Focus group

Seven pharmacists from four companies attended the focus group, which lasted approximately 90 minutes. All had provided the PCP service in their pharmacies and were in the process of arranging the six monthly reviews for their patients. Information emerged relating to twelve of the TDF domains with the only ones not represented being ‘intentions’ and ‘memory, attention and decision processes’. Illustrative quotes aligned with the TDF domains are provided in Table 3 with an explanation of the overarching themes described below.

**Table 3:** TDF domains, illustrative quotes and explanations.

TDF domain	Illustrative quote
**Knowledge**	*“Lifestyle as well*, *I personally felt a little bit out of depth because we’ve never been fully trained on lifestyle… and I was like ok*, *so I go to the gym*, *what did I google myself*, *what did I do myself for myself*, *so like that*.*”* ***Ph6***
**Skills**	*“I cheated you see*, *I got my [relative] to be my first one*. *She’s a brilliant*, *like poorly controlled diabetes*, *so*, *you know*, *she could have benefited from it*, *but that was like*, *having someone who I knew*, *who it didn’t really matter if I screwed up on it*.*”* ***Ph7***
**Social/professional role and identity**	*“Knowing that information [HbA1c] would be useful but*, *like I say*, *at the practice next door*, *if it’s high then they do something about it… So for us to see it would be useful*, *from our perspective but we wouldn’t really do an awful lot with it because the nurse next door is doing*, *the diabetic nurse is dealing with that kind of thing… So it’s kind of duplicating people doing the same thing really*.*”* ***Ph1***
**Beliefs about capabilities**	*“I don’t think I felt fully confident after my first*, *until after my first consultation*. *[Ph1 agrees]*. *That’s why I always explain to the person*, *he was my first so I told him [laughter amongst group]*.*”* ***Ph5*** *“I mean initially*, *occasionally I would get some*, *I mean X would come in and do a little bit [pharmacist cover] if I had a few patients booked in*, *but yes it has been a struggle fitting everything in and like I said*, *managing patients is difficult*.*”* ***Ph3*** *“The problem with the goals was that the goals were anything*, *lifestyle*, *anything*. *If it had been*, *if it’d narrowed the field down and you can sort of like say like it’s going to be diet*, *it’s going to be exercise or whatever maybe you could sort of like focus in on that*, *but some of them were some really…”* ***Ph1***
**Optimism**	*“it got you feeling a bit like motivated at the end of the day*, *like ‘yeh we can do this’*.*”* ***Ph6*** *“it was kind of exciting to be part of something that you know kinda shapes something that could be rolled out in the future”* ***Ph4***
**Beliefs about consequences**	*“…it was like*, *let the patient decide the goals*, *which is fine*, *because you don’t want them to do something you want them to do*, *they’ve got to decide*, *that’s fine*. *But that makes it so broad… So*, *yeh*, *you ended up with any sort of goal*, *from a lot of people just wanting to do the obvious thing like lose weight and like other people who wanted to do like slightly different things… It’s quite broad*.*”* ***Ph2*** *“…we had a lot of emphasis on ‘you have to get patients who’s going to stick with this’ so then I picked kind of really regular customers who always want a chat anyway*, *and that was kind of what I was told to do*. *But then they are not necessarily the people who are going to benefit from it the most [agreement]*.*”* ***Ph7***
**Reinforcement**	*“…my staff initially they were like ‘we’re not getting paid for it*, *so why are we doing it’ but I was like ‘no but you know*, *it’s for human kind’*, *but they don’t even see that do they*?*”* ***Ph6***
**Goal setting**	*“…for the first reviews I would have 10 patients one month and 10 patients the next month*, *so to take that pressure off myself*. *So*, *just to fit in a couple a week isn’t as much as trying to fit in 20 over 2 weeks*.*”* ***Ph3***
**Environmental context and resources**	*“I think even with 20 though*, *that’s 20 hours of work that you’re supposed to fit around” Ph2 “I’d say it was more than 20 hours*.*” Ph7 [agreement around room] “already besides all the work you do reading things at home*, *writing the paperwork on top of everything you already do*. *I think we were quite fortunate because our manager did attend one of the trainings and he was like ‘there’s no way they can do this without double cover’ and we did get double cover for the initial session*. *I think if that wasn’t the case I don’t think I’d have been able to do it*.*”* ***Ph2*** *“The healthcare assistant who I had at the training left shortly after and then you are kind of just stuck really*. *I had to train other members of the staff up without really having the training myself*. ***Ph7*** *“Yeh*, *I really relied on NHS websites*, *so I…[cut off by 7]… 12 week diet plan”* ***Ph3***
**Social influences**	*“I think initially whenever something is new it always takes a bit of time*, *sometimes you have to be the role model to show your team*, *so like you do your first few then let them take over*.*”* ***Ph5*** *“What might have been a good idea is… getting your 10 shops together*, *or 10 pharmacists in a room and let them discuss how to go about it before the consultations start*. *We never had that but I think that would have been a good idea*. *…but that would have been a better sell*, *because if I had been able to talk to 3*, *then we could have gone back and yeh*, *first consultation ‘let’s go for it’ and know roughly how it’s going to work*.*”* ***Ph5*** *“I used ‘so this is a pilot scheme that the NHS is doing*, *you are so special like fit the criteria’*, *like quite a few people went with that because they were like ‘aw*, *it’s for the NHS’*.*”* ***Ph6***
**Emotion**	*“I think they [training team] were a very motivational team*. *They inspired you to do the project*, *and kind of showed us or told us how important*, *we were an important part of the project and that makes you want to do well in it really and*.*”* ***Ph3*** *“It felt great*, *yeh*, *because the time and effort that you’ve put in you’ve actually seen a positive change and that*, *you’ve played a part in that patient making a difference to them*, *because you don’t see them every day*, *but they see the change everyday*, *they see the benefit everyday and if you can do that with every patient you can interact with it’s a good feeling*.*”* ***Ph4***
**Behavioural regulation**	*“If you’ve done it [training for obtaining clinical measurements] then your team do think ‘well the pharmacist can do it’*, *whereas this way*, *I just took a back seat on that one and let my HCA do it and then train everyone else as well so that way they weren’t expecting me to be part of that*, *the metrics as well*.*”* ***Ph6***

#### Training for the service

Pharmacists were positive regarding the consultation skills training which had been provided and recognised the need to practise when they returned to the pharmacy in order to become competent at delivering the service. To address this some pharmacists had undertaken further reading, some had discussed the service with colleagues whilst others reported running a full consultation with a simulated patient, e.g. a relative, before they felt competent. This additional reading and practise was deemed necessary to ensure the pharmacist maintained the patient’s confidence in their ability.

One element of the training that the pharmacists were particularly supportive of was the inclusion of other pharmacy staff. They saw this as having two primary benefits: firstly, it allowed the staff to learn about the service and motivate them to participate when returning to the pharmacy and secondly, it resulted in a better distribution of workload and use of skill-mix when the service was delivered. The need for training additional staff in the pharmacy was identified to provide cover for trained staff absences.

Pharmacists left the training sessions feeling motivated and inspired to provide the service, seeing the benefit that it may potentially have on patients. Some pharmacists also highlighted the effect they felt seeing their staff motivated to provide the service and that other people, aside from just themselves, were engaged with it. With patients starting to return for their follow-up consultations, pharmacists felt positive when discussing the progress they had made with their goals. However, whilst recognising that the PCP service would be valuable for patients, some pharmacists felt as though it was ‘another thing to do’ on top of existing heavy workload.

#### Support and resources

This was a recurrent theme within the focus group and centred on two main elements. Firstly, pharmacists highlighted the time commitment to properly familiarise themselves with the logistics and (core) elements of the service. Many of them described committing extra time outside of their working hours to read the documents associated with the study and practise the consultation. The main motivating factor explaining this extra commitment relates to the earlier point about not wanting to let the patient down in the first consultation because they were not familiar with the relevant procedures.

The second element related to resources centred on having sufficient pharmacist cover to enable provision of the service. All of the pharmacists identified the difficulty with conducting the initial consultations with patients (which in some cases could last up to one hour). They reported techniques to manage their workload by staging appointments over a longer period and asking for locum pharmacist cover to enable them to spend the time with patients. However, this cover was difficult to arrange and variable in availability.

Concerns regarding time commitment and availability of backfill was restricted to the main (longer) consultations. They felt the shorter, interim consultation could be incorporated into routine practice without further resources and support. However, with the additional paperwork and data entry required for this service evaluation, pharmacists also felt that more support could be provided to enable them to complete this in a timely manner. Pharmacists identified that if the service was to be commissioned nationally sufficient resources would need to be provided.

#### Role in patient care

During the discussions, pharmacists highlighted the broad nature of the intervention provided to patients. When they reflected on the goals that patients had set for themselves in the initial consultation, they recognised that most of them were lifestyle related. To some of the pharmacists, this was not particularly welcome as they likened themselves to a ‘weight management service’ rather than being able to utilise their expertise on medicines and knowledge on clinical conditions.

The selection of lifestyle related goals highlighted a training need for some of the pharmacists who didn’t feel confident to respond to such requests for support. As part of this service, innovative approaches to resolving this knowledge deficiency included using trusted websites, other colleagues or thinking about how they lived *their* life in order to provide information to patients.

Pharmacists clearly described encouraging the patient to set their own goals and some reported holding back when it came to goals that they thought were not best supported by this particular service. They described letting the patient lead the goal setting discussion. However, this left some pharmacists feeling lacking in confidence and underprepared for the consultation as they didn’t know what the patient was going to discuss or set as a goal prior to the discussion.

Another element that came through the discussion was around the role of the pharmacist within the wider healthcare team. Some pharmacists felt frustrated that they couldn’t refer patients to other NHS services; they had to go through the medical practice. They viewed this as increasing inconvenience for the patient. There was also discussion around access to medical notes and test results with some pharmacists wanting this information to inform the discussion and goal setting with the patient. Other pharmacists said that although this information might be useful it was the doctor’s place to deal with the test results and they didn’t want to initiate a conflict with the medical practice by ‘checking up’ on their care of the patient. There was also an assumption that if a clinical result was abnormal on medical notes, the doctor would have already taken care of it; a notion which was challenged by other pharmacists within the discussion.

Some pharmacists highlighted that they managed to recruit all their patients on their own and the referral forms were similar to those they would normally use and therefore no additional contact with the medical practice was necessary. Where pharmacists had engaged with the medical practice at an early stage they had patients referred into the service as a result. Those pharmacists felt that they could have more of an effect on these patients as the medical practice had identified them as being in need of the service.

## Discussion

Community pharmacists have so far recruited a large number of patients to the Pharmacy Care Plan (PCP) service over a short period of time. From the analysis of baseline data it is clear that there is potential for community pharmacists to have an impact on the care of these patients as the majority are overweight or obese, have hypertension and high cardiovascular risk. Community pharmacists have demonstrated that they are able to agree a large number of goals with patients and that most of these focus on lifestyle and the desire to improve their condition.

The large number recruited to this new community pharmacy service in a short space of time indicates the willingness of patients to engage with the pharmacist and their team. The recruitment levels are similar to those observed in previous UK community pharmacy service evaluations [25, 30] introduced by the same organisations. Recruited patients were willing to complete the large number of questionnaires at the beginning of the service including those on adherence, patient activation and quality of life and the quality of data collection was excellent which means that a robust service evaluation can be undertaken when the service is completed. Further information on the results of these validated tools will be presented in subsequent papers analysing the effect of the service over the course of the first six months.

From baseline data, the types of patients who were recruited into this study demonstrated that there is a significant proportion of the population who may benefit from an intervention of this nature and that community pharmacists can identify and recruit them. Whilst some of the pharmacists did not believe that weight management is within their remit there is a clear need for interventions to reduce this when the cardiovascular risk and blood pressure in such patients was so high. Losing weight and increasing physical activity are recommended as first line interventions for patients with high cardiovascular risk and are preferable to medicines initiation [14–16]. Being able to signpost patients to such services could enhance the effectiveness of this intervention with the pharmacist playing a monitoring and motivational role when patients collect their medicines.

In general, pharmacists in the focus group appeared motivated and engaged in providing this new service to patients. They identified the importance of the consultations skills training in order to provide a person-centred intervention which may in be part due to a recently released national training package aimed at improving these skills [31]. During the discussion, pharmacists stated that they resisted the need to suggest goals to patients and let patients lead the consultation. It was clear that by allowing patients to set their own goals the pharmacists reported not feeling confident when providing lifestyle advice and some were unsure as to whether this should form part of their professional role. This confusion over professional role is something that has previously been highlighted in the literature and is not uncommon for pharmacists working in the community setting [32].

Whilst the results from this initial evaluation could be used to inform the design of a training package to support pharmacists when providing this service the wide variety of goals which were selected may make this impractical. It may be that the role of the pharmacist is to provide the intervention themselves where possible and to encourage appropriate lifestyle changes and signpost patients to services where they could be better supported and then monitor their adherence to these recommendations. Local public health focussed services commissioned in community pharmacy may be beneficial so that following the identification of patients health goals, patients could be directly enrolled in a service specific for their needs, often delivered by another member of the pharmacy team.

A recurring theme throughout the discussion was the ability to perform the consultation whilst not neglecting their other professional duties. It is not unusual for pharmacists to mention this when questioned by researchers [33–35]. Pharmacists were not remunerated for providing this service and they developed strategies to allow them to perform the extended consultations. This may be due to the novel nature of the study or as some expressed, the desire to forward service development in community pharmacy. However, they did highlight that if implemented nationally, remuneration would be necessary to ensure greater support could be provided. If commissioned, the service would be funded appropriately and therefore these concerns would potentially be overcome.

The general assertion by the group that they didn’t feel able to refer the patient to other services may reflect the independence of the service from other primary care activities. Greater integration of the service with other public health initiatives may have reduced the perception of role ambiguity over what the pharmacists were allowed to do for patients without causing conflict with the wider primary care team. Enhanced interaction with the medical practice would increase their confidence in referring patients for other services and provide the pharmacists with greater definition of their role of patient care.

One element to this interaction with the medical practice surrounds the use of patient data. There is a move to give community pharmacists greater access to medical practice notes which could have two primary benefits for pharmacy service provision. Firstly, the pharmacist could have access to up-to-date information thereby reducing reliance on patient recall for information such as test results. Secondly, it will potentially allow for the appropriate targeting of patients for pharmacy services such as those who are poorly controlled. In previous studies examining GP perceptions of community pharmacy services there has been some criticism of identifying inappropriate patients and duplicating work already performed by the practice [36–38]. With access to patient notes this may have the potential to overcome this problem. Another method of overcoming duplication and role ambiguity is to involve both professions in the training for services, the identification of patients and discussion of care plans. This may help the community pharmacist to identify and have confidence in their role and abilities. It will also help the GP to see what the pharmacist can achieve with a patient under their care.

### Strengths and limitations

This service included a wide range of patients with various health conditions and differing clinical metrics e.g. blood pressure and QRisk2. However, we excluded those with certain diagnoses e.g. myocardial infarction due to the complexity of their condition and management. These patients may have a lower quality of life than those included in the service and this may therefore lead to bias when the results are reported. The results for the clinical metrics also indicate that these patients would not be classified as seriously unwell. However, this is not necessarily a negative as patients with more serious illness are more likely to be managed directly via the GP or hospital. The service also recruited patients who were mostly white, this will therefore limit the generalisability of the results to other populations within the UK.

In terms of the description of the types of goals being set by patients, these figures should be interpreted with caution. From data screening it became apparent that there was variation in the categorisation of the same goals by different pharmacists. In order to ascertain how pharmacists were conducting the service, appropriate fidelity testing would have been useful to determine how pharmacists were implementing the service.

In terms of the focus group, this discussion consisted of a small, convenience sample of pharmacists that were able to be released to attend the session. There was representation from the main four companies involved in the CPF team, however, no pharmacists from the participating supermarkets or independents were able to attend. One risk in obtaining a small sample size is the lack of representativeness of the varying pharmacist viewpoints. The transcript was analysed by two of the authors who were either a pharmacist (MT) or employed by the pharmacy company (CK). This may have had an impact on bias in terms of analysis and selection of quotes; however, both positive and negative comments towards the service were described which indicates that the pharmacists were not selected due to a positive bias towards their employer. Finally, the topic guide and analysis were performed using a theoretical framework derived from the literature and intended to improve the implementation of interventions into routine practice. A COREQ checklist is included in S1 Table.

## Conclusion

In summary, community pharmacists have managed to recruit a large number of patients to a new person-centred service. From baseline data, it appears that they have recruited patients in whom lifestyle and medication-related interventions would potentially have some benefit. In the course of speaking to pharmacists, they described methods of overcoming issues regarding implementation of the service and remained positive as they could see the potential benefit to integrating the role of community pharmacy in the wider primary care team.

There is a move within the NHS to create more person-centred services as many patients now have complex needs associated with multiple conditions. In this new service, the use of agreeing goals was a new way of engaging patients to help support them through making changes to improve their lifestyle and condition management. The focus group identified that pharmacists were positive about the service with respect to both patient benefit and service development and approaches which could be used to enhance service implementation and delivery further. However, pharmacists feel adequate support and appropriate resources are central to successful service provision. Future papers will look at the impact of this new approach in terms of the outcome measures presented here.

## Supporting information

S1 Dataset CPF Paper 1 data for publication v3 Mar 2017. (XLSX)

S1 Table COREQ supplementary file. (DOCX)
