# Supplementary material for: The UK Pharmacy Care Plan service: Description, recruitment and initial views on a new community pharmacy intervention
Source: PLoS One. 2017 Apr 3;12(4):e0174500. doi: 10.1371/journal.pone.0174500 (PMC5378349; doi:10.1371/journal.pone.0174500)
Supplement: S1 Table — (DOCX) [file pone.0174500.s002.docx]

**The UK Pharmacy Care Plan service: description, recruitment and initial views on a new community pharmacy intervention**

**Supplementary file: COREQ checklist.**

**Table 1: COREQ checklist for interviews and focus groups**

| **COREQ category** | **COREQ explanation** | **Response** |
| --- | --- | --- |
| Interviewer/facilitator | Which author/s conducted the interview or focus group? | Detailed in the method. |
| Credentials | What were the researcher's credentials? E.g. PhD, MD | This is detailed in the information for the submitted manuscript. |
| Occupation | What was their occupation at the time of the study? | This has now been included in the method text. |
| Gender | Was the researcher male or female? | This is not relevant for this study as the issues are not gender specific. However, it is clear from the names of the researchers that gender balance was ensured. |
| Experience and training | What experience or training did the researcher have? | Information now added. |
| Relationship established | Was a relationship established prior to study commencement? | No relationship was established prior to study commencement. |
| Participant knowledge of the interviewer | What did the participants know about the researcher? e.g. personal goals, reasons for doing the research | This was explained to the participants via the information sheet and at the start of the focus group. This information is included in the topic guide that can be included as a supplementary file if requested. |
| Interviewer characteristics | What characteristics were reported about the interviewer/facilitator? e.g. Bias, assumptions, reasons and interests in the research topic | The reasons for researching this area are described in the method. The reasons for using the TDF were described. Information relating to the profession of the interviewers has been added. Bias and assumptions are discussed in the strengths and limitations section of the discussion. |
| Methodological orientation and Theory | What methodological orientation was stated to underpin the study? e.g. grounded theory, discourse analysis, ethnography, phenomenology, content analysis | A framework analysis was performed using the Theoretical Domains Framework – both of these were referenced in the data analysis section of the method. |
| Sampling | How were participants selected? e.g. purposive, convenience, consecutive, snowball | A convenience sample was identified and this is stated in the method. The academics had no involvement in the selection of pharmacies, patients or focus group participants. Employers selected pharmacies based on those that were likely to be able to conduct the service successfully. Pharmacists recruited patients as they presented to the pharmacy. Focus group participants were selected by employers based on convenience and the ability to free them from service delivery to attend the feedback session. |
| Method of approach | How were participants approached? e.g. face-to-face, telephone, mail, email | The approach is described in the method. |
| Sample size | How many participants were in the study? | This is included in the method in terms of how many were invited and then in the results in terms of how many participated. |
| Non-participation | How many people refused to participate or dropped out? Reasons? | This was detailed in the results section – reasons were not obtainable from the drop-outs. |
| Setting of data collection | Where was the data collected? e.g. home, clinic, workplace | This focus group was held at a pharmacist feedback event in a hotel meeting room. This is mentioned in the methods. |
| Presence of non-participants | Was anyone else present besides the participants and researchers? | No – we do not believe it to be relevant to include a statement to reflect this in the manuscript. |
| Description of sample | What are the important characteristics of the sample? e.g. demographic data, date | The date of the focus group is detailed in the method and the relevant demographic data is recorded in the results. |
| Interview guide | Were questions, prompts, guides provided by the authors? Was it pilot tested? | This can be provided as a supplement to the paper if requested by the journal. This guide was discussed as a research team prior to the focus group. |
| Repeat interviews | Were repeat interviews carried out? If yes, how many? | No repeat interviews were conducted. |
| Audio/visual recording | Did the research use audio or visual recording to collect the data? | The focus group was audio recorded and this is noted in the method. |
| Field notes | Were field notes made during and/or after the interview or focus group? | Field notes were made but were not used during the analysis phase. |
| Duration | What was the duration of the interviews or focus group? | This information has been added to the results section. |
| Data saturation | Was data saturation discussed? | Data saturation was not possible in this study and is an inappropriate concept given the study design and aims. |
| Transcripts returned | Were transcripts returned to participants for comment and/or correction? | No, we did not think that this would be a useful exercise. |
| Number of data coders | How many data coders coded the data? | This is detailed in the data analysis section of the method. |
| Description of the coding tree | Did authors provide a description of the coding tree? | No, this is not appropriate for this study. |
| Derivation of themes | Were themes identified in advance or derived from the data? | This is explained and referenced in the data analysis section of the method. |
| Software | What software, if applicable, was used to manage the data? | No software was used, the scissors and paste method was used. |
| Participant checking | Did participants provide feedback on the findings? | No – we did not think this would be suitable for this study. |
| Quotations presented | Were participant quotations presented to illustrate the themes / findings? Was each quotation identified? e.g. participant number | Yes – table 3 illustrates these and they are attributed to various participants. |
| Data and findings consistent | Was there consistency between the data presented and the findings? | Yes, this is clear from the text and the table and the inferences drawn from the data are described in the discussion. |
| Clarity of major themes | Were major themes clearly presented in the findings? | Yes – this is clear from both the text and the table. |
| Clarity of minor themes | Is there a description of diverse cases or discussion of minor themes? | Although we have not labelled the themes as major and minor, this is clear from the text and the table and the inferences drawn from the data are described in the discussion. |
